# ISG Task Force on Upper Gastrointestinal Bleeding (UGIB)—Comparison of Risk Scores in Nonvariceal Bleeding—A Prospective Multicenter Study

**DOI:** 10.1002/jgh3.70463

**Published:** 2026-10-06

**Authors:** Manoj K. Sahu, Avinash Balekuduru, Saroj K. Sinha, Varghese Thomas, Manu Tandan, Shrikant Mukewar, Mahesh K. Goenka, Bailuru V. Tantry, Bonthala S. Satyaprakash, Rakesh Kochhar, Rajkumar P. Wadhwa, Philip Augustine, Sujoy Pal, Ebby George Simon, Mathew Philips, V. G. Mohan Prasad, Pravin Rathi, Manas K. Panigrahi, Sri P. Misra, Ajay K. Jain, Vinod K. Mishra, Sanjeev S. Khanna, Kanishka Uthansingh, Govind K. Makharia, Sandeep Nijhawan, Harsh Udawat, Bhabadev Goswami, Krishnadas Devadas, Gautam Ray, Satheesh Rao, Rajiv Baijal, Robert P. Panakkal, Dipak Shah, Abhinav Sharma, Mukesh Agarwala, Sanjay Kumar, Shivaram P. Singh

**Affiliations:** ^1^ Department of Gastroenterology Institute of Medical Sciences & SUM Hospital, SOA Bhubaneswar India; ^2^ Department of Gastroenterology M. S. Ramaiah Medical College Bengaluru India; ^3^ Department of Gastroenterology Postgraduate Institute of Medical Education and Research Chandigarh India; ^4^ Department of Gastroenterology Malabar Medical College Hospital Modakkallur Calicut India; ^5^ Department of Gastroenterology Asian Institute of Gastroenterology Hyderabad India; ^6^ Department of Gastroenterology Midas Multispeciality Hospital Pvt. Ltd. Nagpur India; ^7^ Department of Gastroenterology Apollo Gleneagles Hospital Kolkata India; ^8^ Department of Gastroenterology Kasturba Medical College Mangalore India; ^9^ Department of Gastroenterology Apollo BGS Hospitals Mysore India; ^10^ Cochin Gastroenterology Group Ernakulam Medical Centre (P) Ltd. Kochi India; ^11^ Deptartment of G.I. Surgery & Liver Transplantation All India Institute of Medical Sciences New Delhi India; ^12^ Department of Clinical Gastroenterology & Hepatology Christian Medical College Vellore India; ^13^ Lisie Institute of Gastroenterology Lisie Hospital Kochi India; ^14^ Institute of Gastroenterology VGM Hospital Coimbatore India; ^15^ Department of Gastroenterology Topiwala Nair Medical College and BYL Nair Charitable Hospital Mumbai India; ^16^ Department of Gastroenterology All India Institute of Medical Sciences Bhubaneswar India; ^17^ Department of Gastroenterology & Hepatology Moti Lal Nehru Medical College Prayagraj India; ^18^ Department of Gastroenterology Choithram Hospital & Research Center Indore India; ^19^ Gastro Liver Hospital Swaroop Nagar Kanpur Kanpur India; ^20^ Department of Gastroenterology Holy Spirit Hospital Mumbai India; ^21^ Department of Gastroenterology KIMS Cancer Center, KIMS Hospital Bhubaneswar India; ^22^ Department of Gastroenterology and Human Nutrition All India Institute of Medical Sciences New Delhi India; ^23^ Department of Medical Gastroenterology Sawai Man Singh Medical College and Hospital Jaipur India; ^24^ Department of Gastroenterology Santokba Durlabhji Memorial Hospital (SDMM) Jaipur India; ^25^ Department of Gastroenterology Gauhati Medical College and Hospital Guwahati India; ^26^ Department of Gastroenterology Government Medical College Trivandrum India; ^27^ Gastroenterology Unit, Department of Medicine B.R. Singh Hospital Kolkata India; ^28^ Department of Gastroenterology Narayana Multispecialty Hospital Mysore India; ^29^ Department of Gastroenterology Pushpawati Singhania Hospital & Research Institute New Delhi India; ^30^ Department of Gastroenterology Amala Institute of Medical Sciences Thrissur India; ^31^ Department of Gastroenterology Proton Clinic Kolkata India; ^32^ Department of Gastroenterology and Hepatology Fortis Escorts Hospital Jaipur India; ^33^ Department of Gastroenterology Apollo Hospital Guwahati Guwahati India; ^34^ Gastrocare: Liver & Digestive Disease Center Bhopal India; ^35^ Kalinga Gastroenterology Foundation Cuttack India

**Keywords:** acute non variceal gastrointestinal bleeding, Glasgow Blatchford score, Rockall score

## Abstract

**Background:**

To compare the performance of the Glasgow Blatchford score (GBS) to the Total Rockall score (TRS) in predicting clinical interventions in Indian patients with acute nonvariceal upper gastrointestinal bleeding (ANVUGIB).

**Materials and Methods:**

We prospectively studied ANVUGIB in 93 hospitals in India from October 2014 to September 2018. The etiological, clinical, and investigational details were recorded on a uniform structured proforma. The GBS and TRS were prospectively calculated for all patients. Clinical interventions were defined as blood transfusion, rebleed, and mortality with 30‐day follow‐up.

**Results:**

A total of 6450 patients with ANVUGIB were identified, followed up for 30 days and selected for analysis. Almost 5176 (80%) of the patients were male, and the mean age 50.7 ± 16.8 years (2–98 years). Blood transfusion was considered clinically necessary in 115 (1.7%) of the patients. Surgery and interventional radiology were required as rescue therapy in only 20 patients (0.3%) and 22 (0.3%) patients, respectively. The 30‐day rebleeding and mortality occurred in 92 (1.4%) and in 184 (2.8%) patients, respectively. GBS was similar to TRS in predicting mortality (AUC 0.687 vs. 0.663) with cutoff values of 10.5 and 3.5 and predicting rebleeding with (AUC 0.637 vs. 0.630) cutoff values of 11.5 and 3.5, respectively. Outcomes were defined as 30‐day mortality and rebleeding. Interventions were defined as transfusions, repeat endoscopy, surgery, or interventional radiology.

**Conclusions:**

GBS was more accurate and had more desirable clinical characteristics in predicting the need for a transfusion for hospitalized patients than TRS. However, both GBS and TRS have poor to modest predictive capability for 30‐day mortality and rebleeding. They do not, then, appear to be useful as screening tools to identify people at risk for those two outcomes in a hospital setting.

## 1 Introduction

Nonvariceal upper gastrointestinal bleeding (NVUGIB) is bleeding from the digestive tract proximal to the ligament of treitz [1]. The incidence of NVUGIB is 20–60/100 000 people [2], with 7%–14% mortality [3]. It is a potentially life‐threatening GI emergency with a wide range of clinical severity, ranging from insignificant bleeds to catastrophic exsanguinating hemorrhage [4].

Many risk factors are known to influence the outcome in upper gastrointestinal bleeding (UGIB). Age, comorbidities, presence of shock, endoscopic diagnosis, hemoglobin values at the time of bleed, ulcers' size, stigmata of recent hemorrhage, and need for a blood transfusion have all been described as significant risk factors for rebleeding and death [5, 6, 7, 8, 9].

Thus, it is extremely important to classify NVUGIB severity based on patients' clinical profiles by risk assessment scoring systems. Risk assessment scoring systems have been devised to define the likelihood of death, rebleeding and the need for intervention (e.g., endoscopy or operative surgery).

Total Rockall score (TRS) ranges from 0 to 11 and is based upon age, the presence of shock, medical comorbidity, and a range of endoscopic findings and was developed to define the risk of death, rebleeding, and duration of admission [4]. The Glasgow Blatchford score (GBS) is based upon simple clinical observations, hemoglobin, and blood urea concentrations and, while it is a little more cumbersome to use than the Rockall score, it has the advantage that it can be calculated at an early stage after hospital admission and does not require the endoscopy finding [10].

The TRS and GBS systems are used widely in clinical trials of therapy and their use in clinical decision making in routine practice is less clear. The following study examined the effectiveness and performance values of GBS and TRS scoring systems in clinical application in Indian patients presenting with NVUGIB.

## 2 Materials and Methods

Patients presenting with NVUGIB were prospectively enrolled in the 93 multicenter study from 2014 to 2018. The performance of TRS and GBS was evaluated. The protocol of the study was approved by the institutional ethics committee. Informed consent was obtained from all the included patients. The study was coordinated as part of the task force on gastrointestinal bleeding by the Indian Society of Gastroenterology.

### 2.1 Study Participants

The inclusion criteria for NVUGIB were the following:

1. All the subjects who had presented with acute UGIB manifesting as hematemesis, melena, hematochezia.
2. Bleeding unrelated to varices as confirmed by endoscopy were classified as NVUGIB.

### 2.2 Exclusion Criteria

1. Variceal bleed (esophageal/gastric, ectopic varices, portal gastropathy, or gastric antral vascular ectasia).
2. Patients not giving consent.
3. Incomplete proforma.
4. Pregnant women with GI bleed.

All the patients were managed by initial resuscitation, hemodynamic stabilization, transfusion as required, intravenous proton pump inhibitors, and endoscopy with endotherapy performed once stabilized.

### 2.3 Data Gathering

A checklist consisting of demographic data (age, sex, and socioeconomic status), history of GI bleeding, comorbidities (diabetes, hypertension, cardiovascular disease, liver disease, malignancy), history of smoking/alcohol, medication history (aspirin, nonsteroidal anti‐inflammatory drugs [NSAID], steroid, anticoagulants), and laboratory findings (hemoglobin, blood urea nitrogen—BUN) as well as the source of bleeding according to endoscopy were recorded on a uniform structured proforma.

### 2.4 Outcome

All the patients were followed up for 30 days for primary/secondary outcomes and TRS and Glasgow Blatchford Score (GBS) were calculated. The performance values were calculated for the outcome variables.

Primary outcome was to compare the utility of prognostic scores of TRS and GBS in predicting all‐cause mortality till the end of follow‐up period. Secondary outcome was to measure the utility of the scores in predicting rebleeding/failure of endoscopy/requirement of transfusion. Rebleeding was defined as recurrent vomiting of fresh blood, melena, or both with either shock or a decrease in hemoglobin concentration of at least 2 g/dL after initial successful treatment and after initial stabilization of 24 h. Blood was transfused to maintain hemoglobin ≥ 7 g/dL in nonvariceal bleeding. Failure of endoscopic therapy was defined as inability to control active bleeding during endoscopy or fall in hemoglobin ≥ 2 g% and melena within 48 h of endoscopy requiring either surgery or radiological procedures.

The secondary endpoints would be the clinical interventions that were tracked in our analyses, specifically repeat endoscopy and surgery as interventions resulting from the failure of initial endotherapy.

### 2.5 Statistical Analysis

SPSS software (version 17.0, SPSS, Chicago, IL) was used for all statistical analyses in this study. The clinical demographic and epidemiologic features were analyzed using a descriptive statistical method (mean ± standard deviation, percentage).

To test the discriminative ability of TRS and GBS for predicting the primary and secondary outcomes, receiver operating characteristic (ROC) curves was plotted. The area under receiver operating characteristic (AUROCs) and their 95% confidence interval (95% CI) were calculated. The AUROCs were compared using Youden *J* index. Initial testing utilized preestablished cutoff values (TRS < 2, 3–4, > 5; GBS < 6, > 6), and the Youden *J* index was used to identify the best cutoffs for the study. Because GBS and TRS are integers, the exact Youden cutoff values (e.g., > 10.5) were approximated to the nearest clinically appropriate integer values (e.g., > 10) when reporting data.

### 2.6 Ethics Approval and Consent to Participate

All respondents provided written informed consent before participating in the study. Participation was strictly voluntary and anonymous, with strict confidentiality of personal information assured. During the study period, nine face‐to‐face meetings of the Taskforce were held to monitor progress; challenges encountered during data collection were periodically discussed and resolved. The protocol of the study was approved by individual Institutional Ethics Committees and informed consent was obtained from all study subjects.

## 3 Results

A total of 12 500 consecutive patients with UGIB underwent endoscopy during the study period. Four thousand one hundred and fourteen patients were excluded due to bleeding from esophageal or gastric varices and 1936 due to failure to provide consent, lost for follow‐up during the study, or due to incomplete data. Therefore, a total of 6450 patients were included in the final analysis: NVUGIB. The details of endoscopic findings in the included patients are shown (Figure 1).

**FIGURE 1:**
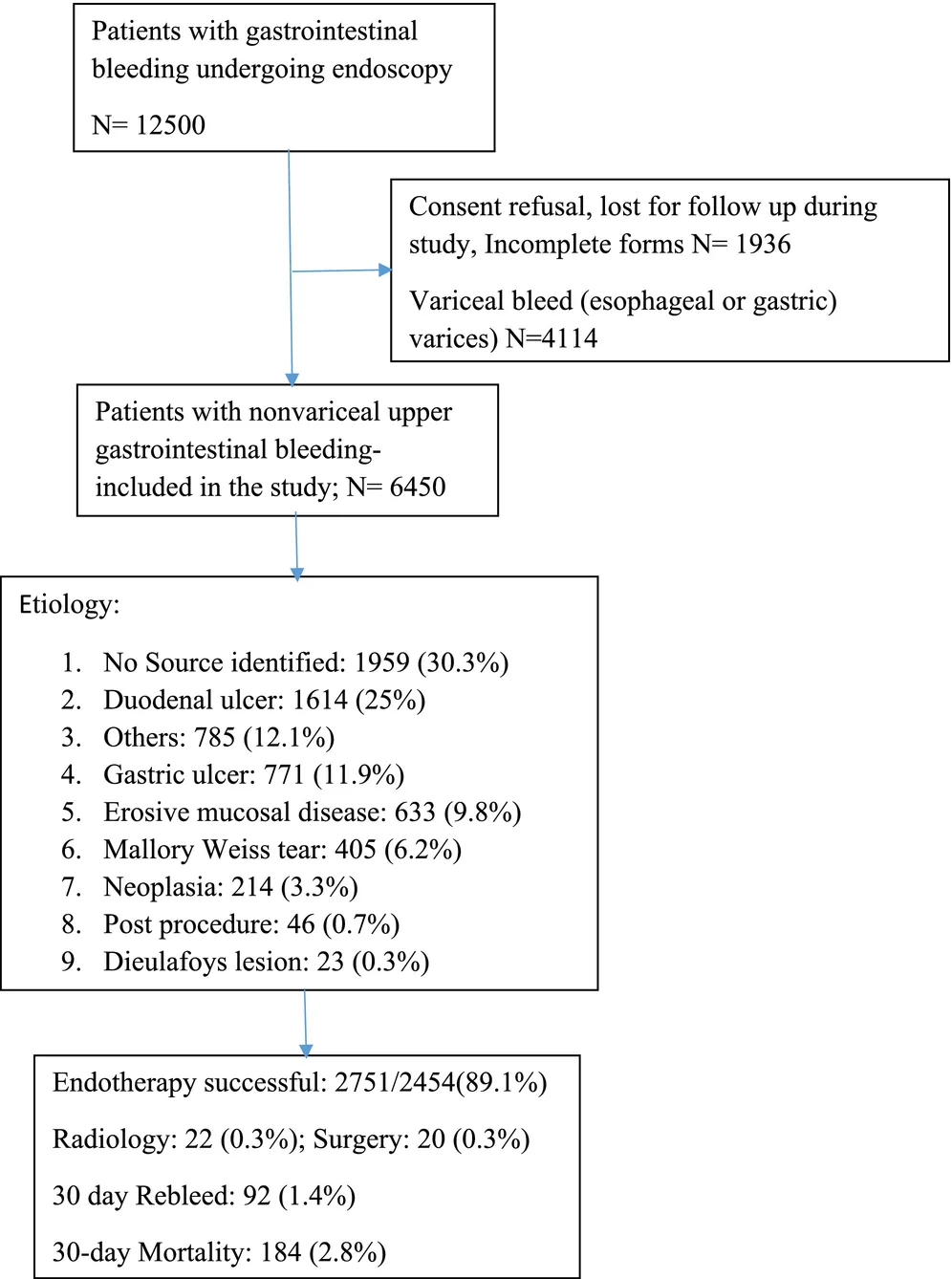
Flow chart of patients recruited in the study.

### 3.1 Characterization of Patients and UGIB Episodes

During the study period, a total of 6450 patients with nonvariceal etiology for UGIB were identified, followed up for 30 days and selected for analysis. Almost 5176 (80%) of the patients were male, and the mean age was 50.7 ± 16.8 years (2–98 years). At admission, melena was the most common presentation of UGIB (in 69.4% patients), while hematochezia was rare (3.7%). Syncope was reported by about one‐tenth of patients. Heart disease and lung disease were the most common comorbidities, being present in 11.9% and 4.3% of the patients, respectively. Regarding medication, antiplatelet agents, NSAID, anticoagulants, and steroids were used in 17.6%, 11.3%, 2.2%, and 1.2%, respectively. Demographic, laboratory results, and scoring system characteristics are shown in Table 1.

**TABLE 1:** Demographic profile of non‐variceal bleeding patients (*N* = 6450).

Sl. no	Features	Features	*n*	%
1	Gender	Male	5176	80.2
		Female	1274	19.8
2	Age in years	< 15	92	1.4
		16–30	824	12.8
		31–45	1527	23.7
		46–60	2145	33.3
		> 60	1862	28.9
3	Socioeconomic status‐Kuppuswamy	Lower	1792	27.8
		Middle	4235	65.7
		Upper	423	6.6
4	Presentation	Hemetemesis	4179	64.8
		Melena	4479	69.4
		Hematochezia	241	3.7
		Syncope	616	9.6
		Abdominal pain	963	14.9
5	Onset of bleed	Outside hospital	5954	92.3
		In patient	496	7.7
6	Comorbidities	Cardiac illness	768	11.9
		Lung disease	278	4.3
		Renal disease	264	4.1
		Musculoskeletal disease	248	3.8
		Hematology problem	78	1.2
		Malignancy	142	2.2
7	Associated drugs	Antiplatelets	1137	17.6
		NSAIDS	731	11.3
		CoX‐2 inhibitors	60	0.9
		Steroids	80	1.2
		Anticoagulants	141	2.2
		Antidepressants	55	0.9
8	Addictions	Current smoker	1380	21.4
		Alcohol intake	1964	30.4
9	Hemoglobin (g/dL)	< 8	2287	35.5
		8–10	2172	33.7
		> 10	1991	30.9
10	Blood urea nitrogen (mg/dL)	< 18	1825	28.3
		19–40	3411	52.9
		41–70	886	13.7
		71–120	318	4.9
		> 120	10	0.2
11	Glasgow Blatchford score	> 6	5392	83.6
		< 6	1058	16.4
12	Total Rockall score	> 5	1783	27.6
		3–4	2197	34.1
		< 2	2470	38.3

### 3.2 Risk Score Calculation

The two risk scores mentioned in the methods were then derived with our collected data. Patients were initially stratified by the traditional cutoff values in order to assess for baseline risk and allow for comparison to descriptive epidemiological reports in the literature. With these cutoffs, 83.6% of our cohort had a high‐risk GBS > 6 and 38.3% of the overall patient population had a low‐risk TRS < 2 (Table 1). The distributions of interventions and outcomes within these descriptive thresholds are shown in Table 2. We then derived an optimal cutoff to better predict the outcome in our patient population for additional ROC analysis.

**TABLE 2:** Diagnostic value of GBS and TRS in predicting mortality, surgery, repeat endoscopy, rebleed, and blood transfusion.

Parameter	GBS < 6	GBS ≥ 6	*p*
Mortality	13 (1.2%)	171 (3.2%)	0.001
Surgery	1 (0.1%)	19 (0.4%)	0.232
Rebleed	8 (0.8%)	84 (1.6%)	0.046
Repeat endoscopy	48 (4.5%)	541 (10%)	0.001
Transfusion	1 (0.1%)	114 (2.1%)	0.001

Parameter	TRS < 2	TRS 3–4	TRS > 5	*p*
Mortality	24 (1%)	79 (3.6%)	81 (4.5%)	0.001
Surgery	3 (0.1%)	9 (0.4%)	8 (0.4%)	0.09
Rebleed	19 (0.8%)	35 (1.6%)	38 (2.1%)	0.001
Repeat endoscopy	142 (5.7%)	230 (10.5%)	217 (12.2%)	0.001
Transfusion	13 (0.5%)	44 (2%)	58 (3.3%)	0.001

### 3.3 Clinical Outcomes and Interventions

We assessed both clinical outcomes (mortality and rebleeding) and clinical interventions (blood transfusion, repeat endoscopy, surgery, and interventional radiology) in NVUGIB. In terms of clinical interventions, 115 patients (1.7%) received blood transfusions, which was deemed medically indicated. Patients received a median of two red blood cell units (IQR: 0–8) transfused. Therapeutic procedures were performed during the primary endoscopy in 2752 patients (42.6%): 2012 patients with injection of adrenaline, 243 patients with hemoclip placement, 290 patients with thermal coagulation, 182 patients with other procedures, and 207 patients with combination therapy. Endotherapy was performed in 2454 patients successfully (89.17%); further procedures were used as second‐line therapy. There was no need for rescue endoscopy. Only 20 patients (0.3%) and 22 (0.3%) patients needed surgery and interventional radiology, respectively. With respect to clinical outcomes, 92 (1.4%) and 184 (2.8%) patients had developed rebleeding and all‐cause mortality at Day 30.

### 3.4 Comparison Between Scores

The diagnostic value of GBS and TRS for predicting clinical outcomes (mortality and rebleeding) and interventions (transfusion, repeat endoscopy, and need for surgery) is depicted in Table 2.

The corresponding AUCs for mortality were 0.68 [95% CI 0.64–0.72] for GBS and 0.66 [95% CI 0.62–0.69] for TRS. The optimal, clinically relevant cutoff for GBS was > 10, with a sensitivity and specificity of 66.3% and 60.5%, respectively. For TRS, the optimal cutoff was > 3 (Figure 2 and Table 3).

**FIGURE 2:**
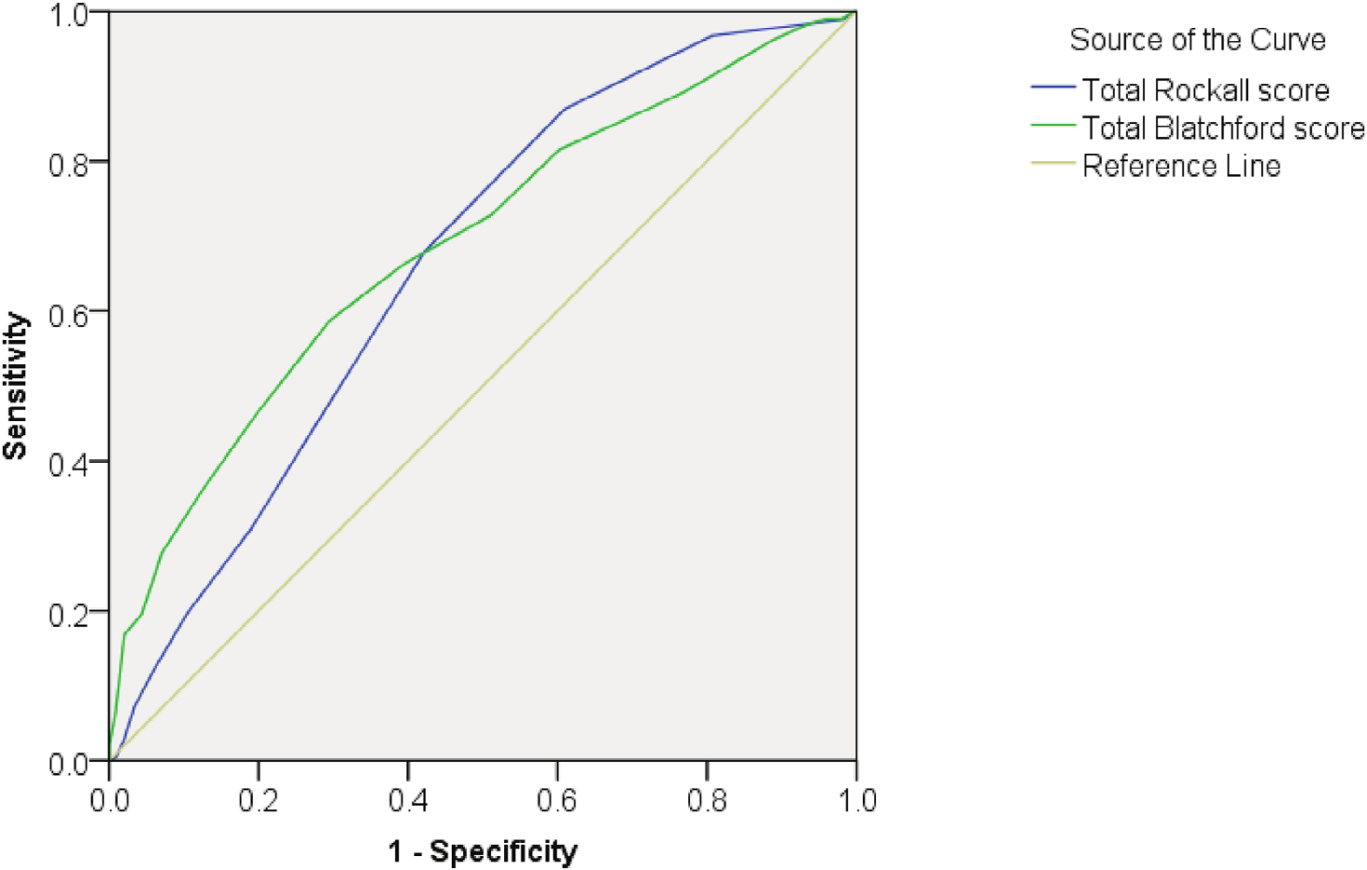
ROC area of GBS and TRS in predicting mortality.

**TABLE 3:** GBS and TRS in predicting mortality with ROC area and cutoff value.

Mortality	Sensitivity	Specificity	ROC area	95% CI	Youden *J* index (cutoff value)
GBS	66.3	60.5	0.687	0.64–0.72	0.26 (10.5)
TRS	67.8	57.8	0.663	0.62–0.69	0.25 (3.5)

The AUCs for rebleeding were 0.63 [95% CI 0.57–0.68] for GBS and 0.63 [95% CI 0.58–0.69] for TRS. The optimal cutoff for both scoring systems was > 11 for GBS and > 3 for TRS (Figure 3 and Table 4).

**FIGURE 3:**
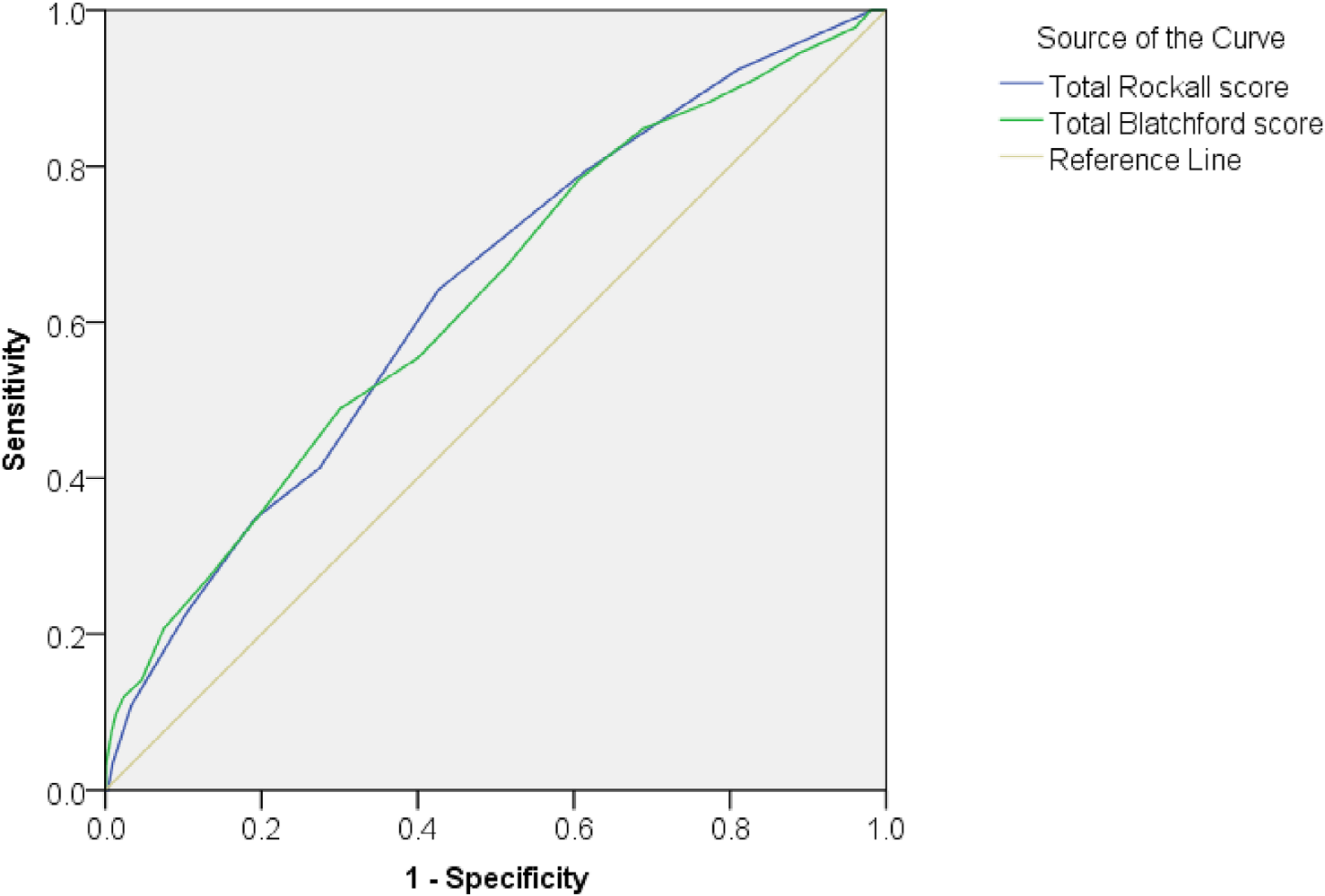
ROC area of Blatchford and Rockall score in predicting rebleeding.

**TABLE 4:** Blatchford and Rockall score in predicting rebleeding with ROC area and cutoff value.

Rebleed	Sensitivity	Specificity	ROC area	95% CI	Youden *J* index (cutoff value)
GBS	48.9	69.9	0.630	0.57–0.68	0.18 (11.5)
TRS	64.1	57.4	0.637	0.58–0.69	0.21 (3.5)

For transfusion, the AUCs were 0.76 [95% CI 0.72–0.80] for GBS and 0.68 [95% CI 0.64–0.73] for TRS, with optimal cutoffs of > 10 for GBS and > 2 for TRS (Figure 4 and Table 5).

**FIGURE 4:**
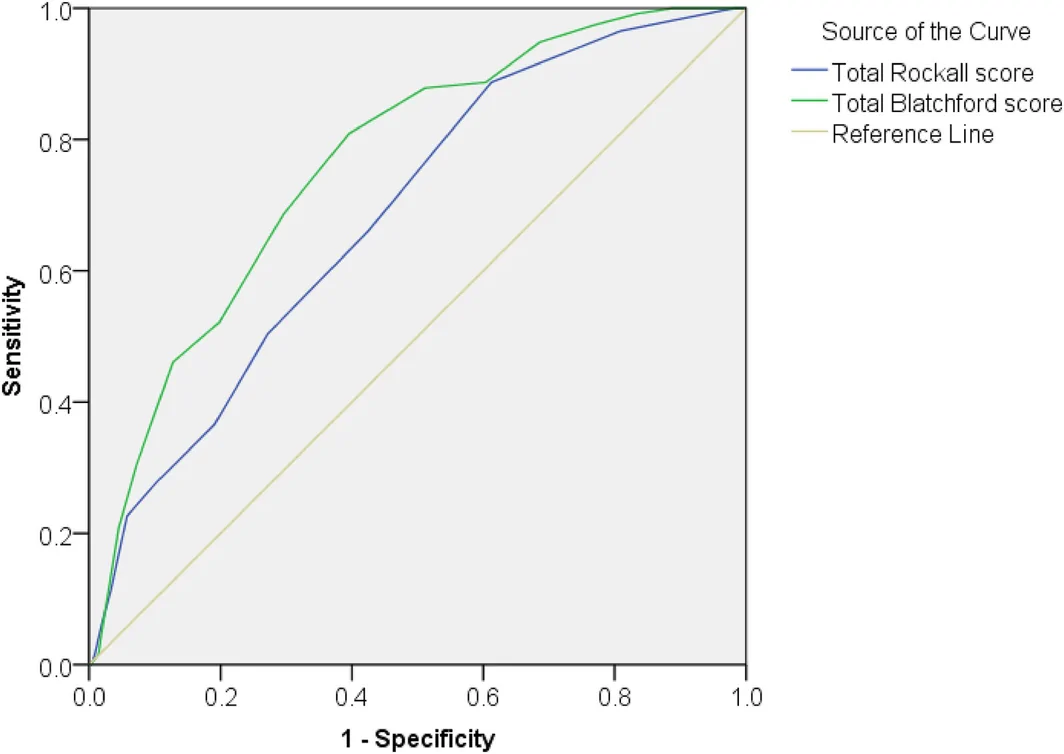
ROC area of Blatchford and Rockall score in predicting transfusion.

**TABLE 5:** Blatchford and Rockall score in predicting need for transfusion with ROC area and cutoff value.

Transfusion	Sensitivity	Specificity	ROC area	95% CI	Youden *J* index (cutoff value)
GBS	80.9	60.5	0.764	0.72–0.80	0.41 (10.5)
TRS	88.7	38.8	0.685	0.64–0.73	0.27 (2.5)

The AUCs for the GBS and TRS to predict rebleeding were 0.63 (95% CI 0.57–0.68) and 0.63 (95% CI 0.58–0.69), respectively (Table 4 and Figure 3).

The AUCs for the GBS and TRS in predicting the need for transfusion were 0.76 (95% CI 0.72–0.8) and 0.68 (95% CI 0.64–0.73) (Table 5 and Figure 4).

## 4 Discussion

UGIB accounts for 25–35 hospitalizations per 100 000 person‐years [11]. The clinical management can be optimized by accurately predicting the outcome. Current guidelines recommend that risk assessment should be performed to triage for timing of endoscopy, patient allocation, time of discharge, and level of care [12, 13].

The scoring systems used to assess risk include GBS and TRS, which use clinical, laboratory, and endoscopic data. TRS and GBS scoring systems are currently used in clinical settings for stratifying the risk in NVUGIB [14]. Nevertheless, their clinical effectiveness remains unclear, and there is little evidence available for the Asian population. In 2011 [15], the Asia–Pacific Working Group consensus on NVUGIB formulated NVUGIB treatment guidelines that were adapted to the Asian population based on the actual conditions of people in the Asia–Pacific region (e.g., economic level and drug metabolism), while also collating research related to NVUGIB risk assessment in the Asia–Pacific region [16]. Unfortunately, there is a lack of large‐sample randomized controlled trials that would provide a higher level of evidence. The aim of the present study was to verify the efficacy of the two scoring systems in clinical application in NVUGIB Indian patients.

In this study, we collectively analyzed the clinical outcomes in 6450 patients with NVUGIB according to GBS and TRS scoring systems. The AUROCs obtained using the GBS and TRS systems were quite comparable for mortality prediction (0.687 vs. 0.663) and rebleeding (0.630 vs. 0.637). GBS had a considerably greater absolute AUROC than TRS (0.764 vs. 0.685) and a more balanced sensitivity and specificity at the ideal cutoff points of 10.5 and 2.5, respectively, when used to anticipate the need for blood transfusion.

This is in concordance with the results from the studies by Laursen et al. [17], Vreeburg et al. [18], Camellini et al. [19], and Robertson et al. [14], concluding that the TRS is not appropriate for prediction of rebleeding.

Our study showed that the GBS is superior to TRS in predicting the needs for blood transfusion. Budimir and colleagues [20, 21], Robertson et al. [14], Custovic [22], Taslidere et al. [23], and Rivieri et al. [24] published similar data.

The GBS scoring system [10], is used to determine outpatient treatment or upon admission to the hospital; early discharge can be done for saving medical resources [25]. In the present study, the predictive value of GBS scoring system for mortality and rebleeding was not satisfactory.

Robertson et al. [14], in their retrospective study of 424 patients, have shown that the Glasgow Blatchford Score (GBS) was superior to the full Rockall score in predicting blood transfusion (AUROC, 0.78 vs. 0.66, *p* < 0.001) but equivalent in predicting the composite endpoint of mortality, rebleeding, and intervention (AUROC, 0.63 vs. 0.62, *p* = 0.82). This was very similar to our findings in a considerably larger patient cohort. Bryant et al. [21] and Custovic et al. [22] proved that the predictive value of the Blatchford score with reference to the need for inpatient transfusion and surgery is superior to the complete Rockall score, but the predictive values for endoscopic intervention, rebleeding, and mortality differed from our research results, which might be due to the inclusion of patients with variceal UGIB.

The interpretation of our findings is limited by the heterogeneity of clinical intervention definitions in global literature, which complicates direct comparisons of risk score performance. Our cohort demonstrated exceptionally low rates of transfusion (1.7%), rebleeding (1.4%), and mortality (2.8%), likely due to a high proportion of low‐risk patients, notably the 30.3% with no identifiable bleeding source. This selection bias, paired with a high endoscopic success rate of 89.17%, likely diluted the composite endpoints and contributed to the instability of the ROC curves for mortality and rebleeding. Such variability underscores the need for standardized outcome definitions to ensure the external validity and predictive accuracy of prognostic models like the GBS and TRS.

In a retrospective study by Taslidere et al. [23], the Glasgow Blatchford Score (GBS) was shown to have superior predictive ability compared with the Rockall score for mortality and transfusion need in patients with UGIB. The GBS had an AUC of 0.901 (95% CI 0.85–0.95) for mortality and 0.921 (95% CI 0.87–0.97) for ICU need, while the Rockall score had an AUC of 0.752 (95% CI 0.68–0.82) for both outcomes. Both scores were equivalent in predicting rebleeding, with an AUC of 0.752 (95% CI 0.68–0.82) for each. This study is in concordance with our study regarding the superiority of GBS in predicting the need for transfusion. But this study has demonstrated the superiority of GBS over the Rockall score in predicting mortality and is inferior in predicting the rebleeding rate. However, this research was done to study the efficiency of the qSOFA score compared with these scores, and it has a rather small sample size of 133 from a single center.

In a cross‐sectional study by Rivieri et al. [24], the Glasgow‐Blatchford score (GBS) demonstrated superior predictive accuracy compared with the Rockall score for determining the need for intervention (including blood transfusion) or in‐hospital death in patients with UGIB. GBS had an AUC of 0.77 (95% CI 0.75–0.80), while the complete Rockall score had an AUC of 0.65 (95% CI 0.62–0.68). These findings suggest that GBS may be a more valuable tool for risk stratification in UGIB patients.

Despite our large cohort of 6450 patients, the observed event rates for mortality (2.8%), rebleeding (1.4%), and transfusion requirement (1.7%) are notably lower than historically published data. This discrepancy suggests a potential selection bias toward lower‐risk cases, as evidenced by the fact that 30.3% of patients had no identifiable bleeding source and 9.8% had only erosive mucosal disease. The inclusion of such a high proportion of low‐risk bleeds likely undermines the predictive validity of the scoring systems in this specific population and contributes to the relative instability of the ROC curves. Furthermore, the high success rate of early endotherapy (89.17%) and the heterogeneity in how clinical interventions are defined across centers may have further diluted the composite endpoints, limiting the ability of the GBS and TRS to reach higher discriminative accuracy.

A key finding of our investigation which requires consideration is the low number of blood transfusions (1.7%), despite admission Hb being < 8 g/dL in 35.5% of the cohort. There are likely a number of reasons for the low transfusion rate.

Firstly, the recommended transfusion protocol was restrictive, and advocated transfusion if Hb was to fall below 7 g/dL [26], as per national guidelines. Since our Hb data was recorded as a single category (< 8 g/dL), it is likely that many patients actually had Hb < 8 g/dL but > 7 g/dL and thus were not required to have a transfusion under the protocol.

Secondly, this was a “real‐world” observational study across 93 hospitals. While all hospitals received the same treatment recommendations and data collection sheets (proformas), it may not be possible to accurately record all supportive therapies used, and in this observational study we have no way of knowing if the protocol (which recommends Hb threshold for transfusion) was being followed. It is possible that there are inaccuracies in blood transfusion being recorded by the registry, with local variation in the blood transfusion thresholds and the availability of blood products. Therefore, the GBS and TRS should be cautiously used in predicting blood transfusion need in this population, given the low transfusion rate observed.

Another key result of our analysis is the change in the optimal cutoff values when compared with the guideline thresholds. Traditionally, the use of a set threshold for GBS > 6 has been a common practice in the selection of high‐risk patients, who required further intervention or admission. When the traditional threshold for the analysis of our cohort, GBS > 6, was used, a huge proportion (83.6%) of patients were categorized as high risk. Although the use of the lower threshold ensures the maximum sensitivity and the safety of the patient as no patient would experience no adverse outcome or require further intervention at the threshold of GBS > 6, the specificity in a real‐world, generalized cohort at this threshold is far too low and leads to overadmission/observation of patients with lesser risk.

On the other hand, with the data‐driven optimal thresholds (GBS > 10 for mortality and transfusion requirement) for the analysis, an acceptable degree of specificity was achieved at the cost of a small extent of absolute sensitivity which might be necessary considering the heavy caseload and limited resources of the health care setting that requires the clinicians to direct the resources (blood transfusion or endoscopy) appropriately at the truly high‐risk patients rather than the whole system at large with a lower cutoff.

Retrospective studies of Chen et al. [27], and Chandra et al. [28], in patients with UGIB showed that GBS identified patients at high risk for clinical interventions (blood transfusion, endoscopic or surgical intervention) better compared with the TRS (92.1% vs. 81.6%, *p* < 0.0001) and (AUC, 0.79 vs. 0.62; *p* = 0.0001) respectively. Stanley et al. [29], prospectively studied 1555 consecutive patients in four UK hospitals. This study showed that GBS outperformed the pre‐endoscopic RS (AUC, 0.944 vs. 0.756; *p* < 0.00005) in predicting the need for transfusion and endoscopic or surgical intervention (AUC, 0.858 vs. 0.705; *p* < 0.00005). But the specificity of both scores was suboptimal at all potential decision thresholds so that it was insufficient to recommend using either score in clinical practice [30]. The difficulty in applying GBS in clinical practice is that it incorporates some subjective parameters, such as cardiac failure and hepatic disease. The severity and the clear‐cut definition of how to evaluate these parameters were not well‐defined in the original score [31]. According to current guidelines on the management of patients with UGIB, patients suspected for UGIB admitted to the hospital an early endoscopy < 24 h should be performed. This is because observational studies do suggest a benefit of endoscopy within 24 h after admission in terms of decreased length of stay and surgical intervention [32, 33]. In our study all the NVUGIB patients had endoscopy within the first 24 h of admission.

However, a selection bias exists because 30.3% of our cohort were not found to have an active site of bleeding and 9.8% only showed an erosive mucosal disease. We postulate that this is due to a relatively higher inclusion of such low‐risk patients in our cohort with a self‐limiting presentation explaining the low rebleeding rate of 1.4% (compared with 7%–14% in prior studies) as well as the low mortality rate of 2.8% (compared with 3%–14% in prior studies). This selection bias might have resulted in an artificially poor predictive performance for both GBS and TRS and is the reason why they performed more poorly in predicting rebleed and mortality.

Thus, this study cannot be interpreted to prove the utility of GBS or TRS across the board. This study further suggests that the utility of these two scoring tools is highly dependent on the acuity of the clinical setting and should perhaps only be applied in lower acuity clinical settings with more self‐limiting presentations, rather than those presenting with potentially life‐threatening organic disease.

Effective endoscopy in 96% of the NVUGIB patients and a lesser number of rescue surgery/rebleed or transfusion requirements in the study cohort might have skewed the statistical scores. We believe that cutoff points are clinically more important and the study results for both GBS and TRS for outcome variables of mortality, rebleed, and transfusion requirement are given. Although our results do suggest that effective endoscopy can outweigh the use of prognostic scores, the evaluation of the patients with UGIB should be guided as per existing guidelines [16].

## 5 Conclusions

Our study has several strengths. This is the first prospective multicenter Indian nonvariceal bleed study showing that a GBS performs better than TRS in the prediction of clinical and endoscopic interventions. The exclusion criteria included patients who did not have upper gastrointestinal endoscopy and had variceal bleeding from the total cohort.

The limitation of this study is that we have applied arbitrary criteria for subjective parameters of GBS which may be different from other studies. Complete Rockall score includes endoscopic findings as a component, thus it cannot be used to triage patients for the need for endoscopy or hospital‐based management. All the patients recruited in our study were of Indian descent. Therefore, the performance of the scores should be validated in other populations. There is always a certain level of subjectivity in the variation in endoscopic treatment, and the study had a relatively short follow‐up period of 30 days. The record of clinical outcomes is limited to the events that happened during the stay at hospital, which could cause bias. All the centers were tertiary care, and there might be a referral bias of the patients.

To conclude, ROC curves analysis showed that GBS demonstrates a higher discriminative tendency and more balanced clinical value for predicting need for transfusion during admission than TRS; both GBS and TRS exhibited only fair predictive value for the outcome measures of 30‐day mortality and rebleeding. Due in part to low sensitivities in predicting rebleeding, neither score is accurate enough to warrant its use as an independent screening test for these events, and clinical judgment as well as early endoscopy must always be the guiding principle for these patients.

## Funding

This work was financially supported by the Zuventus Healthcare Ltd. for project ISG Taskforce on UGIB.

## Ethics Statement

The study protocol was approved by the Institutional Ethics Committees from each center.

## Consent

Written informed consent was obtained from all participants prior to enrollment, and participant confidentiality was strictly maintained throughout the study.

## Conflicts of Interest

The authors declare no conflicts of interest.

## Data Availability

The data that support the findings of this study are available from the corresponding author upon reasonable request.
